# The leafhopper genus *Onukigallia* Ishihara, 1955 with descriptions of two new species from southern China (Hemiptera, Cicadellidae, Megophthalminae, Agalliini)

**DOI:** 10.3897/zookeys.622.9218

**Published:** 2016-10-06

**Authors:** Hu Li, Ren-Huai Dai, Zi-Zhong Li

**Affiliations:** 1 Bio-resources Key Laboratory of Shaanxi Province, School of Biological Sciences & Engineering, Shaanxi Sci-Tech University, Hanzhong, Shaanxi, 723000 P.R. China; 2 Institute of Entomology, Guizhou University, The Provincial Key Laboratory for Agricultural Pest Management of Mountainous Region, Guiyang, Guizhou, 550025 P.R. China

**Keywords:** Auchenorrhyncha, China, distribution, morphology, Onukigallia

## Abstract

Two new Chinese species of the leafhopper genus *Onukigallia*, *Onukigallia neoonukii* **sp. n.** from Sichuan and Guangdong Provinces, and *Onukigallia tumida* **sp. n.** from Hubei and Hunan Provinces are described and illustrated. A key and an updated checklist with distributions of *Onukigallia* species are provided.

## Introduction

The leafhopper genus *Onukigallia* is a small group in the tribe Agalliini of the subfamily Megophthalminae (Hemiptera: Auchenorrhyncha: Cicadellidae) with a distribution in the Oriental region, and well-known by its similarity to the type genus *Agallia* Curtis, 1833, both sharing stout setae on the male subgenital plates but *Onukigallia* differs from the latter in having hairlike setae on the male pygofer and subgenital plates and in the structure of the aedeagus and subgenital plates. It was established by Ishihara (1955) for *Agallia onukii* Matsumura, 1912 (type species). Later, other authors (Viraktamath 1973, 2011; Anufriev and Emeljanov 1988; Zhang and Li 1999; Zhang 2011) proposed new combinations, described new species, provided identification keys and distribution data, and brought the total species number of the genus to five.

The present paper deals with two new species: *Onukigallia neoonukii* sp. n. from Sichuan and Guangdong Provinces, China and *Onukigallia tumida* sp. n. from Hubei and Hunan Provinces, China which are described and illustrated. A key to species is provided for identification, and the checklist is updated with distributions.

## Material and methods

The higher classification of Cicadellidae and morphological terminology used in this work follow Dietrich (2005) and Viraktamath (2011). Leg chaetotaxy follows Rakitov (1998). Examination of *Onukigallia fanjingensis* Zhang & Li follows Li et al. (2014, 2015). The body length is measured from the apex of the head to the end of the forewings and is given in millimeters (mm).

The type material and other material examined are deposited in the Institute of Entomology, Guizhou University, Guiyang, China (GUGC).

## Taxonomy

### Genus Onukigallia

Taxon classificationAnimaliaHemipteraCicadellidae

Ishihara, 1955

#### Type species.

*Agallia onukii* Matsumura, 1912 by original designation.

#### Remarks.

After Viraktamath (2011). This genus is similar to other two Agalliini genera, *Agallia* Curtis, 1833 and *Formallia* Viraktamath, 2011: it closely resembles *Agallia* in having stout setae on the ventral margin of the male subgenital plate but differs in having hairlike setae on the male pygofer side and subgenital plate and in the shapes of the aedeagus and subgenital plates; it is also similar to *Formallia* in body appearance but differs in having setae both dorsally and ventrally on the subgenital plates and also in the shapes of the male pygofer, connective, and aedeagus.

#### Distribution.

Oriental and Palearctic regions: China, Japan, Korea and Russia.

### Onukigallia neoonukii sp. n.

Taxon classificationAnimaliaHemipteraCicadellidae

http://zoobank.org/68AE9AD3-19B8-4301-80E9-2536E59D3D9C

Figures 1–3 , 9–17

#### Measurements.

Body length including tegmina in repose: ♂, 5.32–5.36 mm; ♀, 5.45–5.85 mm.

#### Description.

**Figures 1–8.**
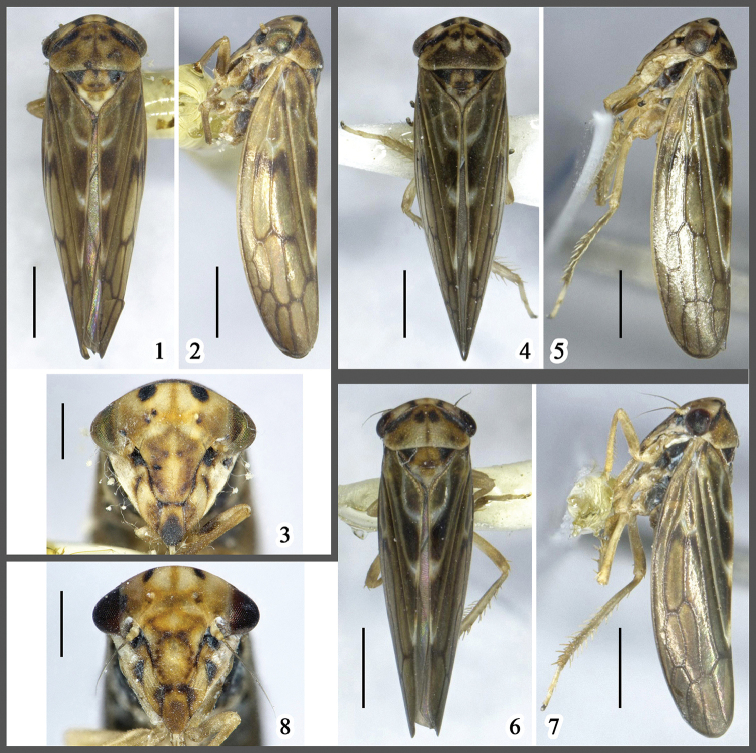
Species of *Onukigallia*, male habitus, dorsal (**1, 4, 6**) and lateral (**2, 5, 7**) views, and face (**3, 8**) **1–3** *Onukigallia neoonukii* sp. n. **4–5** *Onukigallia onukii* (Matsumura, 1912) **6–8** *Onukigallia tumida* sp. n. Scale bars: 1.0 mm (**1–2, 4–7**), 0.5 mm (**3, 8**).

*Body coloration*. Body background color yellowish brown (Figs 1–3). Crown with darker yellowish stripe on midline, and with one pair of black spots separating crown into three equal parts. Face (Fig. 3) upper part with two black spots contiguous with those on crown, midline with yellow stripe extending to base of postclypeus, both sides of midline with white halo; small dark brown macula present ventrolaterad of ocellus; eyes brown, scattered with yellow markings; area below antennal fossa black; lateral frontal suture pale yellow, clypeal sutures dark brown; anteclypeus, distal half black; gena white. Pronotum yellowish brown, anterior margin with black maculae near eyes, midline dark brown, both sides of midline with small and larger paired dark brown maculae. Mesonotum dark brown, lateral angles and basal part of midline black, sides of midline with two small dark brown maculae on distal part. Scutellum with end and lateral angles cream. Forewing claval veins and basal corial veins cream, other veins dark brown. Legs dark brown. Female body color and pattern similar to male but lighter.

*External morphology*. Body appearance (Figs 1–2) typical, slender. Head shorter medially than laterally. Face (Fig. 3) across eyes as long as wide; ocelli transparent, closer to eyes than to each other; anteclypeus round and slightly widened distally, projected beyond lora and gena; transclypeal suture complete. Pronotum nearly 2.0 × wider than broad, oblique frontally and laterally, fore margin prominent, projecting forwards and slightly depressed near eyes, hind margin nearly straight. Scutellum triangular, 1.5 × longer than broad, as long as pronotum. Forewings opaque, venation clearly prominent especially on clavus, with three anteapical and four apical cells, inner anteapical cell closed basally, inner claval vein strongly curved. Hind femoral macrosetae 2+1; hind tibia with 11 macrosetae on PD row, six on AD row, eight on AV row; hind basitarsus with two platellae on distal transverse row.

**Figures 9–19.**
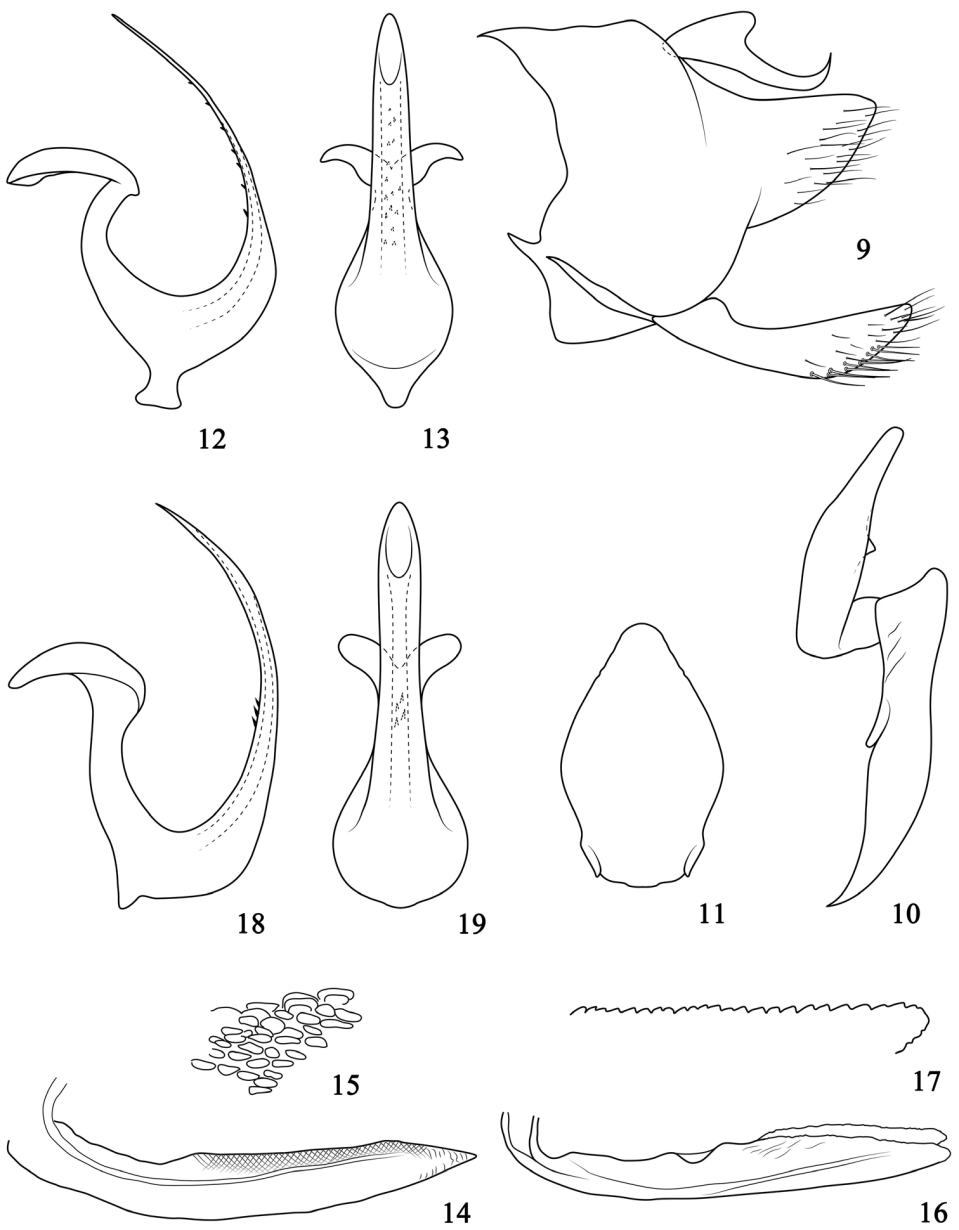
*Onukigallia* species **9–17** *Onukigallia neoonukii* sp. n. **18–19** *Onukigallia onukii* (Matsumura, 1912) **9** Pygofer side and subgenital plate, lateral view **10** Style, dorsal view **11** Connective, dorsal view **12, 18** Aedeagus, lateral view **13, 19** Same, caudal view **14–15** First valvulae **16–17** Second valvulae **15, 17** partial enlarged view.

*Male genitalia*. Pygofer (Fig. 9), basally broad, in lateral view, dorsocaudal and ventrocaudal margins strongly excavated, lobe strongly narrowed and tapering to end, ventrocaudal surface with hairlike setae. Valve wider than long. Subgenital plates (Fig. 9) elongate, slightly exceeding pygofer side, distal half surface with filamentous setae, ventral margin prominent medially and with stout setae in one row. Anal collar process well developed, basally broad, then sharply narrowed and tapered to acute tip twisted dorsally. Style (Fig. 10), robust, inner arm much longer than outer arm, slightly inflated at middle, with small triangular process medially on outer margin, distal half narrowed, end round. Connective (Fig. 11) simple, longer than broad, caudal margin prominent medially, lateral margins expanded near base. Aedeagus (Figs 12–13) broad basally, twisted dorsally, shaft with distal 75% strongly compressed, filiform in lateral aspect, dorsal margin with many small teeth subbasally to subapically, in ventral view, shaft with margin parallel sided, subacute apically; gonopore apical on ventral margin; dorsal apodeme elongate, slender, tip expanded in bilateral direction; preatrium weakly developed.

*Female genitalia*. 7^th^ sternite nearly 1.5 × wider than long, and nearly 2.0 × longer than 6^th^ sternite, hind margin slightly excavated and ridged medially. Ovipositor projecting beyond pygofer. First pair of valvulae (Figs 14–15), in lateral view, relatively broad, slightly curved dorsally from base, tapering, tip sharpened, dorsal half with sculpturing imbricate. Second pair of valvulae (Figs 16–17) dorsally curved slightly from base in lateral view, slightly inﬂated subapically, then tapering to subacute point; dorsal hyaline area (DHA) clear, dorsal prominence (AP) pronounced, apical half of dorsal margin with dense teeth gradually from base to apex.

#### Material examined.

HOLOTYPE: ♂, CHINA: Sichuan Province, Yaan City, Baoxing, Fengtongzhai, 1500 m, 03.VIII.2005, collected by Zhou Zhong-Hui. PARATYPES: 2 ♀♀, Same data as holotype. 1 ♂ 5 ♀♀, CHINA: Guangdong Province, Nanling, Ruyang management station, 850–1500 m, 04–06.VIII.2006, collected by Zhou Zhong-Hui. 4 ♀♀, CHINA: Guangdong Province, Nanling, Longshan Power Station, 500 m, 07–09.VIII.2006, collected by Yang Zai-Hua.

#### Distribution.

China (Sichuan and Guangdong Provinces).

#### Remarks.

The new species is similar to *Onukigallia onukii* (Matsumura) (Figs 4–5, 18–19) but can be distinguished from the latter by the large-sized body (male body length including tegmina in repose of *Onukigallia onukii* is 4.30–4.55 mm), the different color pattern particularly on the pronotum, the more slender aedeagal shaft, and the larger number and wider distribution of teeth on dorsal margin of the aedeagal shaft.

#### Etymology.

The new species name is derived from the Latin words “*neo*-” and “*onukii*”, refers to the similarity to *Onukigallia onukii* (Matsumura).

### Onukigallia tumida sp. n.

Taxon classificationAnimaliaHemipteraCicadellidae

http://zoobank.org/B2BC9011-8481-4C2D-8F10-6E99852A9B8F

Figures 6–8 , 20–24

#### Measurement.

Body length including tegmina in repose: ♂, 4.48–4.50 mm.

#### Description.

*Body coloration and external morphology*. Body (Figs 6–7) relatively darker and more slender than *Onukigallia neoonukii* sp. n. Face (Fig. 8), anteclypeus distal half not black; gena with black macula medially. Pronotum 2.1 × wider than broad. Scutellum with end and lateral angles pale, 1.1 × longer than pronotum and 1.3 × wider than long. Other color pattern and external morphology similar to *Onukigallia neoonukii* sp. n.

**Figures 20–24.**
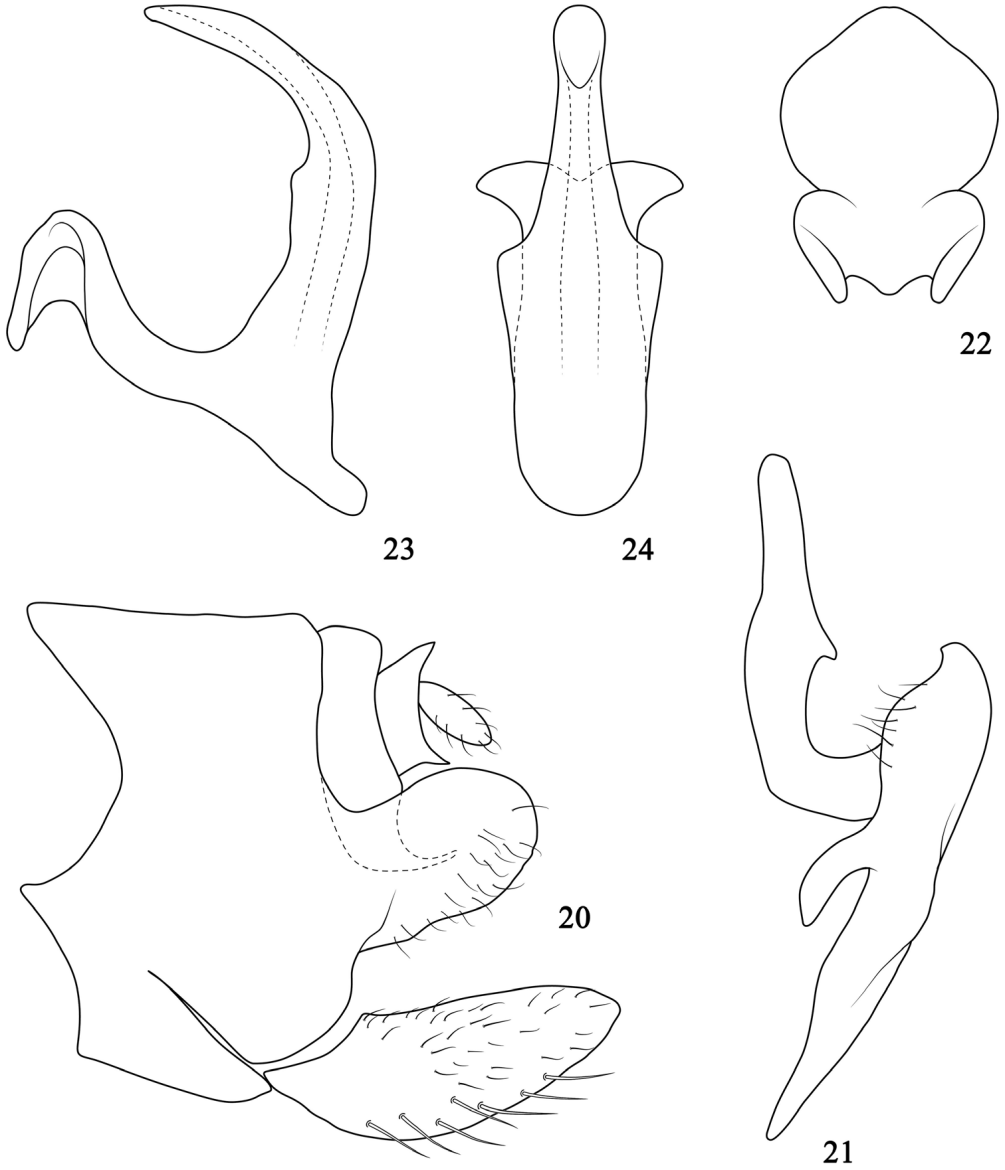
*Onukigallia tumida* sp. n. **20** Pygofer side and subgenital plate, lateral view **21** Style, dorsal view **22** Connective, dorsal view **23** Aedeagus, lateral view **24** Same, caudal view.

*Male genitalia*. Pygofer (Fig. 20), basally broad, in lateral view, lobe strongly narrowed, with dorsal and ventral margins parallel, apex round, ventrocaudal surface of lobe with hairlike setae. Valve broader than long. Subgenital plates (Fig. 20) widened, exceeding pygofer side, surface with filamentous setae, and uniseriate stout setae ventrally. Anal collar process simple, relatively stout, basally broad, then tapered to acute tip twisted caudally. Style (Fig. 21) typical of genus, inner arm 2.0 × longer than outer arm, slightly inflated in middle, with clear triangular process medially on outer margin, apex blunt. Connective (Fig. 22) anterior margin with medial lobe, caudal margin prominent medially, lateral margins slightly expanded near apex, and clearly excavated near base. Aedeagus (Figs 23–24), in lateral view, broad basally, curved dorsally, shaft with small hump near midlength on dorsal margin, ventral margin below gonopore slightly depressed, apex subacute; in ventral view, shaft expanded laterally on lateral margins near base, then tapered, subapex slightly widened and apex round; gonopore apical on ventral margin; dorsal apodeme elongate, tip expanded in bilateral direction.

*Female genitalia*. Unknown.

#### Material examined.

HOLOTYPE: ♂, CHINA: Hubei Province, Shennongjia, 17.VII.2013, collected by Chang Zhi-Min. PARATYPES: 1 ♂, CHINA: Hunan Province, Badagongshan, 03.VIII.2013, collected by Li Hu.

#### Distribution.

China (Hubei and Hunan Provinces).

#### Remarks.

This new species is similar to *Onukigallia fanjingensis* Zhang & Li but can be distinguished from the latter by the different shape of the pygofer lobe and the anal collar process, the aedeagal shaft with an apophysis on its dorsal margin near the base in lateral view, and expanded laterally near base in ventral aspect.

#### Etymology.

The new species name is derived from the Latin word “*tumidus*”, refers to the swollen structure of aegeagal shaft.

### Key to species of *Onukigallia*

Based on the original descriptions, illustrations, and examinations of specimens the following key (largely based on the male genitalia) distinguishes the species of *Onukigallia* except *Onukigallia tenuis* (Matsumura) which is only known by its female.

**Table:** 

1	Aedeagal shaft with teeth on dorsal margin	**2**
–	Aedeagal shaft without teeth on dorsal margin	**3**
2	Aedeagal shaft slender and with several teeth on dorsal margin near middle	***Onukigallia onukii* (Matsumura)**
–	Aedeagal shaft strongly slender and with more teeth on dorsal margin from subbasally to subapically	***Onukigallia neoonukii* sp. n.**
3	Aegeagal shaft with hump on dorsal margin in lateral view near midlength, and expanded laterally near base in ventral view	***Onukigallia tumida* sp. n.**
–	Aegeagal shaft without apophysis on dorsal margin in lateral view, and not expanded laterally in ventral view	**4**
4	Preatrium of aedeagus elongate; pronotum darkly pigmented	***Onukigallia arisana* (Matsumura)**
–	Preatrium of aedeagus short and poorly developed; pronotum paler	**5**
5	Aedeagus lacking preatrium; anal collar process with inflated subapex and round tip	***Onukigallia matsumurai* Zhang**
–	Aedeagus with weakly developed preatrium; anal collar process with tapered subapex and acute tip	***Onukigallia fanjingensis* Zhang & Li**

### Updated checklist and distributions of species of *Onukigallia*

**Table:** 

Species name	Distribution
*Onukigallia arisana* (Matsumura, 1912)	China (Taiwan)
*Onukigallia fanjingensis* Zhang & Li, 1999	China (Shaanxi, Hubei, Guangxi, Guizhou, Fujian, Anhui, Zhejiang)
*Onukigallia matsumurai* Zhang, 2011	China (Yunnan)
*Onukigallia neoonukii* sp. n.	China (Sichuan, Guangdong)
*Onukigallia onukii* (Matsumura, 1912)	China (Gansu, Shaanxi, Ningxia, Shanxi, Liaoning, Jilin, Hebei, Henan, Anhui, Hubei, Hunan, Zhejiang, Guizhou, Sichuan, Yunnan), Japan, Korea, Russia
*Onukigallia tenuis* (Matsumura, 1912)	China (Taiwan)
*Onukigallia tumida* sp. n.	China (Hubei, Hunan)

## Supplementary Material

XML Treatment for Onukigallia

XML Treatment for Onukigallia neoonukii

XML Treatment for Onukigallia tumida
